# TikTok and frozen shoulder: a cross-sectional study of social media content quality

**DOI:** 10.1186/s10195-024-00805-y

**Published:** 2024-11-24

**Authors:** Riccardo D’Ambrosi, Enrico Bellato, Gianluca Bullitta, Antonio Benedetto Cecere, Katia Corona, Angelo De Crescenzo, Valentina Fogliata, Gian Mario Micheloni, Maristella Francesca Saccomanno, Fabrizio Vitullo, Andrea Celli, SICSeG (Italian Society of Shoulder, Elbow Surgery) Research Committee

**Affiliations:** 1 IRCCS Ospedale Galeazzi—Sant’Ambrogio, Milan, Italy; 2 Department of Biomedical Science for Health, University of Milan, Milan, Italy; 3 Department of Surgical Science, University of Turin, Turin, Italy; 4 San Luigi Gonzaga Hospital, Orbassano, Turin, Italy; 5 CTO Andrea Alesini, Rome, Italy; 6 Ospedale San Giuliano, Giugliano, Naples, Italy; 7 Department of Medicine and Health Sciences “Vincenzo Tiberio”, University of Molise, Campobasso, Italy; 8 Ente Ecclesiastico Ospedale Generale F. Miulli, Acquaviva delle Fonti, Bari, Italy; 9 UO Chirurgia della Spalla, Cliniche Humanitas Gavazzeni e Castelli, Bergamo, Italy; 10 University of Modena and Reggio Emilia, Modena, Italy; 11 Department of Medical and Surgical Specialties, Radiological Sciences, and Public Health, University of Brescia, Brescia, Italy; 12 Department of Bone and Joint Surgery, Spedali Civili, Brescia, Italy; 13 University La Sapienza, Rome, Italy; 14 Department of Orthopaedic and Traumatology Surgery, Shoulder and Elbow Unit, Hesperia Hospital Modena, Modena, Italy

**Keywords:** TikTok, Social media, Reels, Frozen shoulder, Adhesive capsulitis, Arthroscopy, Physical therapy, Rehabilitation

## Abstract

**Purpose:**

This study aimed to assess the validity and informational value of the material provided on TikTok regarding frozen shoulders. The hypothesis was that the video content on this platform would not provide adequate and valid information.

**Methods:**

The current study focused on frozen shoulder videos on the TikTok social media platform. The terms “frozen shoulder” and/or “adhesive capsulitis” were used as keywords for an extensive online search of video content on TikTok, and the first 100 videos were included. Out-of-topic, non-English, and duplicated videos were excluded from the analysis. The duration and numbers of likes, shares, and views were recorded for each video. Further, videos were categorized based on the source (physiotherapist/osteopath, medical doctor, or private user), type of information (physical therapy, etiopathogenesis, anatomy, clinical examination, patient experience, or symptoms), video content (rehabilitation, education, or patient experience/testimony), and the presence of music or a voice. The assessment of the video content’s quality and reliability was performed by two experienced shoulder surgeons using the DISCERN instrument, the Journal of the American Medical Association (JAMA) benchmark criteria, and the Global Quality Score (GQS).

**Results:**

A total of 100 videos were included in the analysis, of which 86 (86.0%) were published by physiotherapists/osteopaths. Most of the information and video content focused on physical therapy and rehabilitation (83.0% and 84.0%, respectively). Eighty-four (84.0%) videos included voice comments, while the remaining featured music. The mean number of views was 2,142,215.32 ± 6,148,794.63, while the mean numbers of likes, comments, and shares were 58,438.67 ± 201,863.54, 550.81 ± 1712.22, and 3327.43 ± 7320.81, respectively. The mean video duration was 110.20 ± 116.43 s. The mean DISCERN score, JAMA score, and GQS were 16.17 ± 2.36, 0.61 ± 0.51, and 1.18 ± 0.41, respectively. Videos posted by medical doctors or private users received higher scores than those posted by physiotherapists/osteopaths (*p* < 0.05).

**Conclusions:**

The educational value of videos published on TikTok was poor; videos posted by medical doctors exhibited better quality and educational value than those of physiotherapists or osteopaths. It is the responsibility of orthopedic surgeons to investigate the potential benefits, consequences, and implications of TikTok video content for the health of frozen shoulder patients and to propose necessary adjustments. Given the rapid growth of TikTok, further research is needed.

***Level of evidence*:**

Level IV—cross-sectional study.

## Introduction

Adhesive capsulitis, commonly referred to as "frozen shoulder," is a condition that affects approximately 2% to 5% of the population [1]. The prevalence may be greater due to the disorder's tendency to manifest as a relatively minor and self-limiting ailment [2]. Consequently, a significant number of individuals with this condition may not seek medical intervention [3]. Glenohumeral joint fibrosis is a pathological condition characterized by the formation of fibrous tissue in the glenohumeral joint, resulting in restricted active and passive ranges of motion (ROMs), joint capsule rigidity, and shoulder pain [1]. The illness was initially documented by Simon-Emmanuel Duplay, who coined the term "scapulohumeral periarthritis" [4]. Earnest Codman is credited with creating the term "frozen shoulder" in 1934, highlighting the significant reduction in shoulder mobility that afflicts individuals with the condition [4]. In 1945, Julius Neviaser provided a new definition for the ailment, referring to it as adhesive capsulitis. His detailed examination of the tissue revealed the presence of inflammation and fibrotic alterations in the capsule and nearby bursa. Frozen shoulder is a type of shoulder condition characterized by stiffness. It is commonly considered an idiopathic disorder, meaning that its cause is unknown [4]. The categories of stiff shoulders include postoperative stiffness, posttraumatic stiffness, and neurological stiffness, which typically require distinct treatments and have different prognoses compared to frozen shoulders [5]. Frozen shoulder is generally regarded as a self-limiting phenomenon that typically resolves itself after 1 to 3 years. However, symptoms may persist for a longer duration in approximately 20% to 50% of individuals [5]. There are nonoperative treatment alternatives that can potentially reduce the duration of the condition. For frozen shoulder patients who do not respond to nonoperative treatments, open arthroscopic release is a reliable and efficient therapeutic option [5].

New internet technologies can enhance health communication and patient education [6]. The internet has transformed the role of patients from passive recipients of information to proactive information seekers. Consumers of general health information use social media platforms to seek both practical guidance and psychological support [6]. Although social media offers significant advantages, it also has drawbacks when used for health communication [6]. The literature extensively discusses concerns over the quality of information. The likelihood of encountering erroneous health information on social media platforms enhances the vulnerability of patients, who may base their health decisions on incorrect information [7]. The unfiltered nature of information presents challenges for both patients and healthcare providers. Patients must be able to differentiate reliable sources of information from unreliable ones. Conversely, health professionals and organizations are responsible for addressing and counteracting health misinformation to safeguard the general public. Therefore, it is crucial to scrutinize the quality of health information disseminated on social media platforms [7–10].

This study aimed to assess the validity and informational value of the material provided on TikTok regarding frozen shoulders. The hypothesis was that the video content on this platform would not provide adequate and valid information.

## Methods

The current study was exempt from institutional review board approval.

**Fig. 1:**
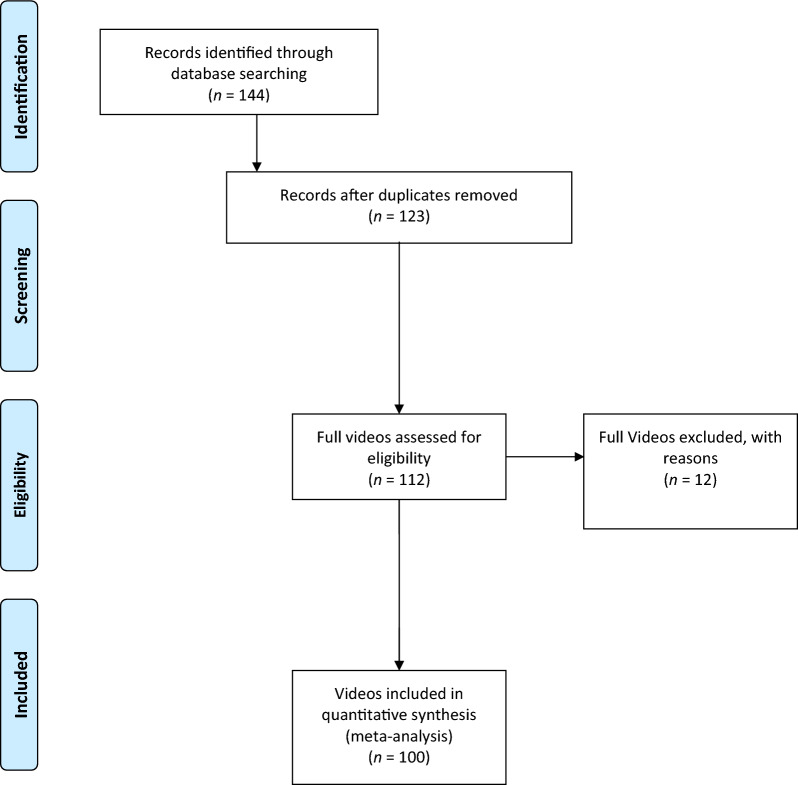
A flowchart of the video screening performed in this study

This study focused on frozen shoulder videos on the TikTok social media platform. The terms “frozen shoulder” and/or “adhesive capsulitis” were used as keywords for an extensive online search of video content on TikTok (November 22, 2023), and the first 100 videos were included. Out-of-topic, non-English, and duplicated videos were excluded from the analysis. Figure 1 presents a flow chart of the video selection process [11].

The duration and the number of likes, shares, and views were recorded for each video. Further, videos were categorized based on the source (physiotherapist/osteopath, medical doctor, or private user), type of information (physical therapy, etiopathogenesis, anatomy, clinical examination, patient experience, or symptoms), video content (rehabilitation, education, or patient experience/testimony), and the presence of music or a voice.

The assessment of the video content’s quality and reliability was performed by two experienced shoulder surgeons using the DISCERN instrument, the Journal of the American Medical Association (JAMA) benchmark criteria, and the Global Quality Score (GQS) [12–14].

### Assessment tools for video reliability, validity, and quality

- DISCERN instrument

The DISCERN tool is an assessment scale developed for patients and providers to evaluate the reliability and quality of information. It consists of 16 items divided into three parts. Items 1 to 8 form the first part and measure the reliability of the information. Items 9 to 15 form the second part, which measures the quality of the information, and the last section consists of a single item with an overall quality rating (item 16). The DISCERN score is determined using a 5-point Likert scale. For the first 15 items, 1 point indicates “no” and 5 points indicate “yes;” the responses are evaluated within this range. For the 16th item, a score of 1 point indicates “low quality with serious or extensive deficiencies,” while a score of 5 points indicates “high quality with minimal deficiencies;” the responses are evaluated within this range. The total DISCERN score is calculated as the sum of the first 15 items, with a minimum score of 15 and a maximum of 75. The reliability and quality of the information are characterized by an increase in scores, where a score of 15–27 points indicates “very poor,” 28–38 points indicates “poor,” 39–50 points indicates “medium,” 51–62 points indicates “good,” and a score of 63–75 points indicates “excellent” reliability and quality [13]. The DISCERN tool is freely accessible at http://www.discern.org.uk.

- JAMA benchmark criteria

The JAMA benchmark criteria instrument is one of the leading tools for evaluating medical information from online sources. It includes four criteria (authorship, attribution, disclosure, and currency), with a value of 1 point each and a total possible score of 4 points. In the JAMA evaluation, a score of 0–1 point represents insufficient information, 2–3 points represent partially sufficient information, and 4 points represent completely sufficient information [14].

- Global Quality Score

The GQS scoring system, developed by Bernard et al., can be used to assess a video concerning its instructive aspects for viewers. This system enables the evaluation of quality, streaming, and ease of use for the information presented in an online video. In the GQS evaluation, a score of 1 indicates that the video has the poorest quality and is not useful for viewers, while a score of 5 indicates excellent quality, so it is very useful for viewers [12].

### Statistical analysis

Descriptive statistics were presented for all video characteristics, including the video source, video content, type of information, and outcomes (i.e., the DISCERN score, JAMA score, and GQS). Categorical variables were shown as absolute frequencies and percentages. The normality of continuous variables was tested using the Shapiro‒Wilk test, and variables were presented as the mean and standard deviation or the median and interquartile range (IQR).

Correlations between quantitative variables were estimated and tested through the Spearman rank correlation test. To assess whether the outcomes differed by video source, video content, audio, and type of information, we first performed Wilcoxon–Mann‒Whitney tests and obtained multiple quasi-Poisson regression models. A regression model was obtained for each outcome using video source, video content, type of information, and video characteristics as independent variables. Quasi-Poisson regression models were selected due to evidence of overdispersion. For the aforementioned analyses, some categories of video sources (doctor and private user), video content (education and patient experience/testimony), and type of information (etiopathogenesis, anatomy, clinical examination, patient experience, and symptoms) were grouped into one category due to their low frequencies.

All tests were two-tailed, and a *p* value of < 0.05 was considered statistically significant. All statistical tests were performed with R (R Foundation for Statistical Computing, Vienna, Austria; https://www.R-project.org/).

## Results

A total of 100 videos were included in the analysis, of which 86 (86.0%) were published by physiotherapists/osteopaths. Most of the information and video content concerned physical therapy and rehabilitation (83.0% and 84.0%, respectively). Eighty-four (84.0%) videos included voice comments, while the remaining featured music. Detailed results are reported in Table 1.

**Table 1:** Categorical variables

	*n* (%)^a^
Video source	
Physiotherapist osteopaths	86 (86.0)
Doctor	13 (13.0)
Private user	1 (1.0)
Company	0 (0.0)
Type of information	
Physical therapy	83 (83.0)
Ethiopathogenesis	9 (9.0)
Anatomy	3 (3.0)
Clinical examination	3 (3.0)
Patient experience	1 (1.0)
Symptoms	1 (1.0)
Video content	
Rehabilitation	84 (84.0)
Education	15 (15.0)
Patient experience/testimony	1 (1.0)
Audio characteristics	
Voice	84 (84.0)
Music	16 (16.0)

^a^ *N* = 100 videos were included in the analysis

The mean number of views was 2,142,215.32 ± 6,148,794.63, while the mean numbers of likes, comments, and shares were 58,438.67 ± 201,863.54, 550.81 ± 1712.22, and 3327.43 ± 7320.81, respectively. The mean video duration was 110.20 ± 116.43 s.

The mean DISCERN score, JAMA score, and GQS were 16.17 ± 2.36, 0.61 ± 0.51, and 1.18 ± 0.41, respectively. Detailed results are reported in Table 2.

**Table 2:** Continuous variables

	Mean ± SD^a^	Median [IQR; range]^a^
Video characteristics		
Total number of views	2,142,215.32 ± 6,148,794.63	370,500.00 [25225–1150000; 213–45900000]
Total number of likes	58,438.67 ± 201,863.54	5758 [489.75–23225; 2–1700000]
Total number of comments	550.81 ± 1712.22	69 [14.50–350.50; 0–14500]
Total number of shares	3327.43 ± 7320.81	370 [50.50–2251.25; 0–39000]
Video length (seconds)	110.20 ± 116.43	65 [35.75–150; 6–600]
Video score		
DISCERN	16.17 ± 2.36	15 [15–16; 15–30]
JAMA	0.61 ± 0.51	1 [0–1; 0–2]
GQS	1.18 ± 0.41	1 [1–1; 1–3]

*IQR* interquartile range,* GQS* Global Quality Score

^a^ *N* = 100 videos were included in the analysis

### Correlations

As shown in Table 3, the scores demonstrated significant positive correlations with each other (*p* < 0.05).

**Table 3:** Correlations between scores

	DISCERN		JAMA	
	*ρ*	*p* value	*ρ*	*p* value
JAMA	0.55	< 0.001*	–	–
GQS	0.70	< 0.001*	0.40	< 0.001*

*N* = 100 videos were included in the analysis;* ρ* was estimated using Spearman’s rank correlation

^*^ Statistically significant value

*GQS* Global Quality Score

### Correlations between scores and video features

The DISCERN score showed a positive correlation with the number of comments (*p* < 0.05); the JAMA score showed positive correlations with the number of shares, number of comments, and video length (*p* < 0.05); and the GQS showed negative correlations with the number of views and number of likes (*p* < 0.05). Detailed results are shown in Table 4.

**Table 4:** Correlations between scores and video features

	DISCERN		JAMA		GQS	
	*ρ*	*p* value	*ρ*	*p* value	*ρ*	*p* value
Total number of views	0.01	0.962	0.08	0.449	− 0.24	0.018*
Total number of likes	0.04	0.677	0.13	0.209	− 0.22	0.027*
Total number of shares	0.18	0.079	0.24	0.016*	− 0.06	0.541
Total number of comments	0.21	0.037*	0.21	0.041*	− 0.04	0.685
Video length (s)	0.16	0.120	0.36	< 0.001*	0.01	0.945

*N* = 100 videos were included in the analysis;* ρ* was estimated using Spearman’s rank correlation

^*^Statistically significant value

*GQS* Global Quality Score

### Correlations between video features

Positive correlations were found between the number of views and the number of likes and number of comments; between the number of shares and the number of views, number of likes, and number of comments; and between the number of likes and number of comments (*p* < 0.05). Detailed results are shown in Table 5.

**Table 5:** Correlations between video features

Variables		*ρ*	*p* value
Total number of views	Total number of likes	0.97	< 0.001*
Total number of shares	Total number of views	0.86	< 0.001*
Total number of shares	Total number of likes	0.86	< 0.001*
Total number of likes	Total number of comments	0.84	< 0.001*
Total number of views	Total number of comments	0.81	< 0.001*
Total number of shares	Total number of comments	0.80	< 0.001*
Total number of comments	Video length (seconds)	0.12	0.252
Total number of likes	Video length (seconds)	0.10	0.305
Total number of shares	Video length (seconds)	0.09	0.393
Total number of views	Video length (seconds)	0.05	0.637

*N* = 100 videos were included in the analysis;* ρ* was estimated using Spearman’s rank correlation

^*^Statistically significant value

### Analysis of the video source

Videos posted by medical doctors or private users received higher scores than those posted by physiotherapists/osteopaths (*p* < 0.05). Detailed results are shown in Table 6.

**Table 6:** Score differences between video sources

	Physiotherapist osteopaths	Doctor or private user^a^	*p* value
	*N* = 86	*N* = 14	
DISCERN	15.52 ± 0.98	20.14 ± 4.04	< 0.001*
JAMA	0.56 ± 0.50	0.93 ± 0.47	0.018*
GQS	1.08 ± 0.28	1.79 ± 0.58	< 0.001*

^a^ Categories were collapsed due to the low frequency of private users as video sources (*n* = 1)

^*^Statistically significant value

*GQS* Global Quality Score

### Analysis of information type

Videos reporting information regarding the etiopathogenesis, anatomy, clinical examinations, patient experiences, and symptoms received higher scores than those reporting physical therapy information (*p* < 0.05). Table 7 depicts detailed results.

**Table 7:** Score differences between types of information

	Physical therapy	Other ^a^	*p* value
	*N* = 83	*N* = 17	
	Mean ± SD	Mean ± SD	
DISCERN	15.51 ± 0.98	19.41 ± 4.00	< 0.001*
JAMA	0.54 ± 0.50	0.94 ± 0.43	0.005*
GQS	1.06 ± 0.24	1.76 ± 0.56	< 0.001*

^a^Ethiopathogenesis, anatomy, clinical examination, patient experience, and symptoms were grouped into one category due to the low frequency of each

^*^Statistically significant value

*GQS* Global Quality Score

### Video content analysis

Videos about education/patient experiences received higher scores than those about physical therapy (*p* < 0.05). Table 8 shows the detailed results.

**Table 8:** Score differences between video content

	Rehabilitation	Education or patient experience/testimony^a^	*p* value
	*N* = 84	*N* = 16	
	Mean ± SD	Mean ± SD	
DISCERN	15.51 ± 0.98	19.62 ± 4.03	< 0.001*
JAMA	0.55 ± 0.50	0.94 ± 0.44	0.008*
GQS	1.07 ± 0.26	1.75 ± 0.58	< 0.001*

^a^Categories were collapsed due to the low frequency of patient experience/testimony (*n* = 1)

^*^Statistically significant value

*GQS* Global Quality Score

### Analysis of the presence of music/voices

No differences were found between videos with voices or music (*p* > 0.05). Detailed results are shown in Table 9.

**Table 9:** Score differences between videos with voices or music

	Voice	Music	*p* value
	*N* = 84	*N* = 16	
	Mean ± SD	Mean ± SD	
DISCERN	16.33 ± 2.53	15.31 ± 0.60	0.091
JAMA	0.64 ± 0.51	0.44 ± 0.51	0.142
GQS	1.21 ± 0.44	1.00 ± 0.00	0.050

*GQS* Global Quality Score

## Discussion

The main findings of our study demonstrated that videos posted on TikTok regarding adhesive capsulitis were published primarily by physiotherapists and osteopaths, with a focus on rehabilitation. Most videos exhibited low educational value, while those posted by medical doctors showed higher educational value across all scores (JAMA score, GQS, and DISCERN score).

Currently, though scientific literature regarding new social media platforms such as TikTok is growing, there is a paucity of research on orthopedic diseases and pathologies.

All available articles present results similar to ours, with very low scores and educational value. Tabarestani et al. recently assessed the quality and educational benefits of Achilles-tendinopathy-related TikTok videos and found that although TikTok is a powerful tool for information distribution, the educational value of the videos related to the specific subject was poor, with only 1% of them receiving a grade of “fair,” and none receiving a score of “good” or “excellent” [15].

Anastasio et al. assessed the quality and educational benefits of ankle-sprain-related TikTok videos. The authors concluded that although TikTok is a powerful tool for information distribution, the educational value of the videos related to ankle sprain injury exercises was poor, with only 2% of videos receiving a grade of “fair,” and none being categorized “good” or “excellent” [16].

Bethell et al. assessed the quality, reliability, and educational benefits of the information presented in videos on shoulder instability exercises on TikTok. The DISCERN scores of videos uploaded by general users had significantly lower scores in all four categories than those uploaded by healthcare professionals. Concerning the shoulder stability exercise education score, videos uploaded by general users had significantly lower scores than those uploaded by healthcare professionals. More general user videos were graded as very poor (84.2%) compared to healthcare professional videos (51.5%). However, the remaining healthcare professional videos were graded as poor (48.5%) [17].

Aflatooni et al. analyzed posts on TikTok to better understand the scoliotic patient experience. A large number of posts were positive (*p* < 0.001) and made by female users (*p* < 0.001). Self-image was the most prevalent subject, with many posts not mentioning activities of daily living, incisional scars, imaging, pain, physical therapy, timing, awareness/education, or the spine levels involved. This may represent a positive public attitude toward scoliosis; however, further research is needed [18].

One factor that differentiates adhesive capsulitis from other conditions is its significant influence on psychological and psychiatric conditions. As seen in the literature, there is also a correlation between the use of social media such as TikTok and the risk of developing depression and anxiety [19–22].

A recent German retrospective cohort study investigated the association between adhesive capsulitis and the 5-year cumulative incidence rate of depression among adults. Within 5 years of the index date, 17.5% of patients with adhesive capsulitis and 8.7% of those without it were diagnosed with incident depression (*p* < 0.001). This result was corroborated by Cox regression analysis, which revealed a positive and significant association between adhesive capsulitis and the cumulative incidence of depression [19–22].

Chiaramonte et al. found a significant correlation between primary frozen shoulder and particular personality traits, indicating an interaction between psychological and somatic factors [23].

Beyond the COVID-19 pandemic, TikTok has implications for public health. According to a TikTok platform search, the hashtags "medicine" and "doctor" received 1.4 billion and 6.7 billion views, respectively [24].

The use of video-based platforms for medical education, including Vimeo and YouTube, is not a recent development. YouTube has been employed to globally disseminate educational videos and clinical expertise on specific subjects. It is well suited for in-depth discussions on topics, as the average YouTube video runs for slightly more than 11 min. Nevertheless, this may limit the learner's ability to remain actively engaged. In contrast, TikTok, a social media platform emphasizing video content, concentrates on videos limited to 60 s in duration. In response to the growing prevalence of "rapid-fire"-style lectures in national conferences and residency programs, TikTok encourages active learning by offering more concise and condensed content. Just as it is imperative to select appropriate material for a rapid-fire lecture meticulously, the subjects of a 60-s TikTok video must also be chosen carefully [25].

TikTok is especially favored by adolescents and youth, serving as the quintessential platform for this demographic. The mean age of Facebook and Twitter users is approximately 40 years, that of Instagram users is approximately 30 years, and the mean age of TikTok users is approximately 20 years, with 40% of users aged 10 to 19 years. This age difference is noteworthy because younger demographics have unique media consumption patterns and are often less susceptible to traditional advertising in conventional media, which this audience has essentially forsaken. Hence, TikTok presents a substantial opportunity for influencer marketing campaigns due to its remarkable expansion, distinctive format, content attributes, and ability to directly engage with youth who have significant consumer potential [26].

Currently, medical education uses social media platforms, including Twitter and Instagram, to facilitate the active sharing of instructional blogs and podcasts, disseminate evidence-based medicine, and provide direct explanations of clinical concepts. Nevertheless, structured data regarding the use of TikTok for this particular objective are scarce. Educators currently employ this platform as a novel approach to enhance medical education for both the general public and other medical professionals [27].

There are limitations to this study. Geographic location and user attributes can potentially impact the outcomes of the search algorithm. The analysis eliminated videos not in English, diminishing the generalizability of the current findings. The present study additionally employed reliability, validity, and quality evaluation instruments, namely, the DISCERN score, JAMA score, and GQS, which have not been completely validated. Nevertheless, these tools are extensively employed in research endeavors for assessing the efficacy of these metrics for online services.

## Conclusions

The educational value of videos published on TikTok is poor; videos posted by medical doctors present better quality and educational value than those posted by physiotherapists or osteopaths. Orthopedic surgeons are responsible for investigating the potential benefits, consequences, and implications of TikTok videos for the health of frozen shoulder patients and proposing necessary adjustments. Given the rapid growth of TikTok, further research is needed.

## Data Availability

Raw data are available upon request to the corresponding author.
